# Enhancing the Quality of Diamond Film Growth Through the Synergistic Addition of Nitrogen and Carbon Dioxide

**DOI:** 10.3390/ma19010183

**Published:** 2026-01-04

**Authors:** Zhanpeng Sheng, Xuejian Cui, Lei Zhao, Yihan Lv, Rongchen Zhang, Defang Kon, Nan Jiang, Jian Yi, Lingxia Zheng

**Affiliations:** 1 College of Chemical Engineering, Zhejiang University of Technology, Hangzhou 310000, China; shengzhanpeng202322@nimte.ac.cn (Z.S.); cuixuejian@nimte.ac.cn (X.C.); 2 State Key Laboratory of Advanced Marine Materials, Zhejiang Key Laboratory of Extreme-environmental Material Surfaces and Interfaces, Ningbo Institute of Materials Technology and Engineering, Chinese Academy of Sciences, Ningbo 315201, China

**Keywords:** MPCVD, synergistic effect, nitrogen doping, defect engineering

## Abstract

This study investigates the synergistic effects of co-doping with ultralow-concentration nitrogen and trace carbon dioxide on the growth of polycrystalline diamond films via microwave plasma chemical vapor deposition (MPCVD). The films were characterized using scanning electron microscopy, X-ray diffraction, Raman spectroscopy, and photoluminescence spectroscopy. Results indicate that trace nitrogen effectively promotes <111> oriented growth and enhances the deposition rate, whereas excessive nitrogen leads to the formation of defects such as pores and microcracks. The introduction of CO_2_ suppresses the formation of nitrogen-vacancy-related defects through a selective etching mechanism. Under co-doping conditions, diamond films with high growth rates, strong <111> texture, and superior thermal conductivity (up to 1863.94 W·m^−1^·K^−1^) were successfully synthesized, demonstrating significant potential for thermal management applications in high-power integrated circuits.

## 1. Introduction

Diamond films possess exceptional hardness, outstanding thermal conductivity, a broad optical transmission window, and excellent chemical stability. These remarkable properties make them promising candidates for a wide range of applications in fields such as mechanical processing, optical windows, semiconductors, and thermal management [1,2]. Chemical vapor deposition (CVD) has become the method of choice for fabricating high-quality diamond films [3]. However, conventional CVD processes, particularly those employing hydrogen and methane as the primary gas precursors, often face challenges in producing high-quality diamond films. The performance of polycrystalline diamond—an aggregate of micron- or nano-sized diamond grains sintered together—is dictated not only by the intrinsic hardness of the diamond crystallites but more critically by the quality of intergranular bonding, the grain size distribution, and the purity of the grain boundaries. Typically, PCD films suffer from microstructural defects such as highly disordered grain orientations, the presence of excessive impurities or non-diamond carbon (e.g., graphite, amorphous carbon) at the grain boundaries, and non-uniform grain sizes [3,4,5,6,7,8]. In MPCVD, the diamond growth kinetics are dictated by the underlying plasma chemistry, itself determined by a set of parameters including gas composition, process pressure, gas flow rate, and substrate temperature [9,10,11,12,13,14,15].

To optimize the growth quality of diamond films, the use of additive gases has been widely adopted as a well-established strategy. Introducing nitrogen gas (N_2_) is a common approach, which has been proven to effectively enhance the growth rate and modify the crystal morphology. However, the sole incorporation of nitrogen often introduces an excessive number of defects, such as nitrogen-vacancy (NV) centers, leading to unstable electrical properties and even a darkening of the film, which compromises its performance in optical applications [15]. Yang et al. [15] successfully synthesized highly textured polycrystalline diamond via ultra-low-level nitrogen doping, achieving rapid growth rates (~3–8 μm/h) and high infrared transmittance (~49.2–70.8%) across a broad spectrum of 3.0–15.0 μm. In contrast, Ashkinazi et al. [16] employed a microwave plasma CVD process with a high-concentration CH_4_-H_2_-N_2_ atmosphere, demonstrating that the morphology of the diamond film grown on a single-crystal substrate was strongly dependent on the substrate’s crystal orientation. However, a critical paradox emerges from these doping strategies. Analysis by scanning electron microscopy and Raman spectroscopy reveals that although nitrogen doping enhances the growth rate, an excessively high rate can induce detrimental microstructural defects, such as surface microcracks and intergranular voids. These defects, in turn, degrade the crystallinity and ultimately compromise the optical performance of the film. The incorporation of oxygen into the process provides a source of highly reactive oxygen atoms (O) and hydroxyl radicals (OH). These species preferentially etch sp^2^-bonded carbon by reacting with it to form volatile carbon monoxide (CO) and carbon dioxide (CO_2_), which are then evacuated from the system. In MPCVD, this is typically achieved by introducing gases such as carbon dioxide (CO_2_) [17]. This mechanism functions as an in situ etching process, which continuously and selectively suppresses the growth of graphitic phases and other defects during diamond deposition, thereby purifying the growth environment and ultimately yielding higher-quality diamond films [18,19]. The work by Gu et al. [20] showed that high-level oxygen doping, particularly at elevated pressures, significantly boosts the etching efficiency of graphite, thereby producing diamond films of higher purity and greater thickness. This enhanced growth was accompanied by an increase in deposition rate and resultant surface roughness. A key microstructural outcome was the substantial increase in the density of (111) facets on the film surface facilitated by the high-pressure conditions. In their study on boron-doped diamond films synthesized via microwave plasma chemical vapor deposition, Zhao et al. [21] observed that as the oxygen concentration (O/C ratio) increased from 0% to 5%, the film’s growth rate initially decreased and then increased. The introduction of oxygen was found to suppress boron incorporation, improve crystal quality, and significantly inhibit residual nitrogen. Hall effect measurements confirmed that at an O/C ratio of 3%, a high-mobility, high-quality diamond film was achieved with a growth rate of 9 μm/h. This work suggests that trace Carbon dioxide can compensate for the crystal strain induced by boron doping, thereby enhancing both crystalline and surface quality. Although a moderate Carbon dioxide addition can enhance film purity and crystallinity by selectively etching amorphous carbon and graphitic phases formed during growth, excessive etching introduces defects. Specifically, if the CO_2_ concentration becomes too high, even the diamond phase undergoes significant etching. This leads to a roughened growth surface, increased defect density, and grain boundary formation, ultimately degrading the film quality. While moderate Carbon dioxide doping enhances diamond film purity and crystallinity by selectively etching amorphous carbon and graphitic phases, excessive etching, particularly from high CO_2_ concentrations, becomes detrimental. It not only attacks the diamond phase itself—leading to a roughened surface, increased defects, and grain boundaries that degrade quality—but also disrupts the optimal CVD growth environment. A hydrogen-rich atmosphere is crucial, as atomic hydrogen stabilizes sp^3^ bonds and passivates surface dangling bonds. The introduction of excessive CO_2_ can upset this balance by consuming active hydrogen species (e.g., forming H_2_O or OH), thereby shifting the growth-etch equilibrium unfavorably and potentially promoting the deposition of non-diamond carbon films. Given the inherent limitations of single-gas additives in precision and efficacy, the synergistic effect of co-introducing gases presents a significant yet underexplored avenue.

This study proposes an innovative multi-component co-doping strategy that achieves precise modulation of diamond crystal facet competition by synchronously introducing trace amounts of carbon dioxide into a ppm-level nitrogen doping system during microwave plasma chemical vapor deposition (MPCVD). This approach effectively addresses longstanding technical challenges in conventional nitrogen-doped polycrystalline diamond fabrication, including disordered crystal orientation, lattice distortion, suboptimal crystallinity, and low thermal conductivity.

Research demonstrates that the introduction of carbon dioxide, through mechanisms such as selective etching of non-diamond phases and regulation of surface reaction kinetics pathways, significantly suppresses the proliferation of crystal defects induced by single-element nitrogen doping. This enables synergistic modulation rather than mere enhancement of the nitrogen doping effect. The nitrogen-doped polycrystalline diamond films fabricated under varying carbon dioxide flow parameters exhibit outstanding comprehensive performance: a highly <111>-oriented crystal structure is successfully achieved, accompanied by a growth rate of approximately 3–4 μm/h and a high thermal conductivity of 1863.94 W·m^−1^·K^−1^, while also offering advantages of short fabrication cycles and relatively low energy consumption.

This work systematically elucidates the dynamic balancing mechanism and crystal quality optimization principles of carbon dioxide within the co-doping system. The developed high-performance diamond films demonstrate significant potential for engineering applications in the thermal management of high-power integrated circuit chips operating under high-speed and extreme thermal conditions.

## 2. Materials and Methods

Free-standing diamond films were synthesized using a microwave plasma chemical vapor deposition (MPCVD) system, where a plasma was generated under strong electromagnetic fields to facilitate diamond deposition. The source gases were hydrogen (H_2_, 99.999%, Linde, Shanghai, China) and methane (CH_4_, 99.999%, Linde, Shanghai, China). Nitrogen concentration was controlled by introducing an H_2_-N_2_ mixture (1% N_2_/99.9% H_2_, Linde, Shanghai, China). A 2-inch diameter, 3 mm thick, p-type doped single-crystal silicon wafer with a (100) orientation served as the substrate. The CH_4_ to H_2_ flow ratio was fixed at 3:100, with the CH_4_ flow rate maintained at 12 sccm for a growth duration of 40 h. The nitrogen concentration during deposition was regulated by introducing 0–1.6 sccm of the H_2_-N_2_ mixture into the reaction chamber. The formula for calculating nitrogen concentration (relative to H_2_) is:

N_2_(ppm) = MN flow rate × 1% ÷ (H_2_ flow rate + MN flow rate × 99%) × 10^6^ (1)

The substrate temperature was kept at 850 ± 10 °C, monitored by an infrared pyrometer (IR-AHS0, Chino, Japan), and the chamber pressure was set at 100 Torr. The relevant parameters are summarized in Table 1.

Substrate pretreatment is a critical step for the heteroepitaxial growth of diamond films, primarily aimed at surface cleaning, nucleation enhancement, and improved film adhesion. A three-step pretreatment process was employed on the silicon substrate: 1. Ultrasonic Cleaning: The substrate was ultrasonically cleaned in absolute ethanol to remove surface contaminants such as organic residues and dust particles, thereby preventing them from adversely affecting the epitaxial quality of the diamond film; 2. Nanodiamond Seeding and Secondary Ultrasonication: A colloidal suspension of W_10_ nanodiamond powder in ethanol was uniformly drop-coated onto the substrate surface. Subsequently, the sample was immersed in acetone and subjected to a second ultrasonic treatment. This step served to further purify the substrate surface and pre-deposit nanodiamond particles, which act as nucleation centers; 3. Mechanical Abrasion: The substrate surface was continuously rubbed in a unidirectional manner with a lint-free cloth. This mechanical action created uniform micro-scratches and embedded nanodiamond seeds, significantly increasing the density of nucleation sites. The treated substrate exhibits a clean surface with a uniform defect distribution, which effectively enhances the adhesion between the diamond film and the substrate. Insufficient adhesion may lead to film delamination or cracking during growth or subsequent processing, resulting in epitaxial failure. Therefore, substrate pretreatment serves as a critical guarantee for high-quality heteroepitaxial diamond growth. After baking in dry air, the silicon wafer was placed into a laboratory-developed disc-shaped resonant MPCVD (GMP60KIPT400, with a maximum power of 6 kw) chamber for further processing. The diamond film was then deposited according to the process parameters listed in Table 1. The heating and cooling rates for the sample were controlled at approximately 15 °C/min. Following the growth process, the sample was cooled down and subsequently removed from the chamber. To obtain a free-standing diamond film, the silicon substrate was selectively etched away using an HF/HNO_3_ mixture (with a volume ratio of HF:HNO_3_ = 3:1). Finally, the resulting diamond film was thoroughly rinsed with deionized water and dried.

The crystal orientation was analyzed using X-ray diffraction (XRD, Advance D8, Bruker, Billerica, MA, USA) with filtered Cu K-α radiation (λ = 1.5406 Å). The measurement was conducted at room temperature in continuous scanning mode, with a 2θ range of 30° to 130. The crystal orientation was analyzed using X-ray diffraction (XRD, Advance D8, Bruker, USA) with filtered Cu K-α radiation (λ = 1.5406 Å). The measurement was conducted at room temperature in continuous scanning mode, with a 2θ range of 30° to 130°, a step size of 0.02°, and a scanning speed of 2°/min. Phase identification was performed by referring to the Powder Diffraction File (PDF) database of the International Centre for Diffraction Data (ICDD), a step size of 0.02°, and a scanning speed of 2/min. Phase identification was performed by referring to the Powder Diffraction File (PDF) database of the International Centre for Diffraction Data (Specific card number: PDF#06-0675) [22]. Furthermore, the surface morphology and microstructure of the diamond films were examined by scanning electron microscopy (SEM, Regulus 8230, Hitachi, Tokyo, Japan).

To enable further investigation, the growth surface of the free-standing diamond film was mirror-polished using a polishing machine (Model: SPG40-II, Supower, Nanjing, China) with commercial diamond powder (median particle size ~2.5 μm) as the abrasive medium. The thermal properties of the diamond material were subsequently characterized using a laser flash analyzer (NETZSCH LFA 467, Yokohama, Japan).

## 3. Result and Discussion

### 3.1. Diamond Film Growth Rate and Its Quality

To investigate the film growth rates under different process conditions, the thicknesses of the free-standing diamond films were measured before and after deposition. The growth rate was calculated based on the thickness difference. As summarized in Table 2, the introduction of nitrogen into the process gas significantly enhanced the growth rate of the diamond film. Specifically, the growth rate increased by a factor of 3.1, from 1.13 μm/h to 3.48 μm/h [23,24].

In the MPCVD growth of diamond thin films, surface hydrogen desorption is the rate-determining step. A pristine diamond surface is passivated by hydrogen atoms. Growth species (e.g., CH_3_ radicals) must locate an active site (a dangling bond) on the surface that is not covered by hydrogen to facilitate bonding. In the absence of doping, the density of active sites (dangling bonds) is very low because hydrogen atoms are strongly bound (strong C–H bond) and difficult to desorb, which severely limits the deposition rate. When nitrogen gas (N_2_) is introduced and activated into reactive nitrogen species in the plasma, nitrogen atoms become incorporated into the surface layer of the growing diamond film. As revealed in the referenced study, the formation of C–N groups alters the local charge and bonding environment [25]. These C–N sites can serve as energetically more favorable “reaction platforms” compared to pristine C–H sites. Specifically, the C–H bonds near C–N sites are weakened, effectively lowering the activation energy barrier for hydrogen desorption—the rate-determining step. Additionally, C–N groups act as dominant active sites, which can “shorten the migration path of CH_2_ and other radicals.” In the context of MPCVD, this implies that carbon-containing growth species adsorbed on the surface can more rapidly locate stable bonding positions (such as another active site or a step edge), thereby reducing the chance of re-passivation by hydrogen before bonding occurs and improving the efficiency of carbon incorporation into the diamond lattice. These combined effects collectively contribute to a marked increase in the film’s deposition rate [26,27,28].

**Figure 1:**
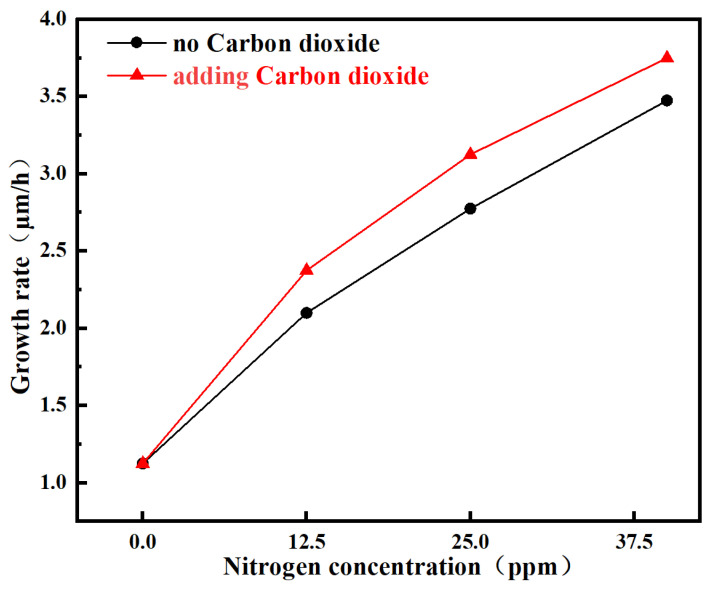
Growth rates of diamond films under different nitrogen gas flow rates before and after adding Carbon dioxide.

Similarly, as shown in Figure 1,the introduction of carbon dioxide (CO_2_) also led to an increase in the film growth rate at the same nitrogen concentration. This enhancement is attributed to the provision of a more efficient carbon decomposition pathway by Carbon dioxide atoms (O**·**) and hydroxyl radicals (OH**·**) derived from CO_2_. The representative reactions, CH_4_ + O**·**→ CH_3_**·** + OH**·** and CH_4_ + OH**·** → CH_3_**·** + H_2_O, typically proceed at higher rates than those involving H**·**. Consequently, under identical power and methane concentration, the gas-phase concentration of reactive methyl radicals (CH_3_) is significantly elevated. This abundance of key growth precursors directly accelerates the diamond deposition rate [20].

**Figure 2:**
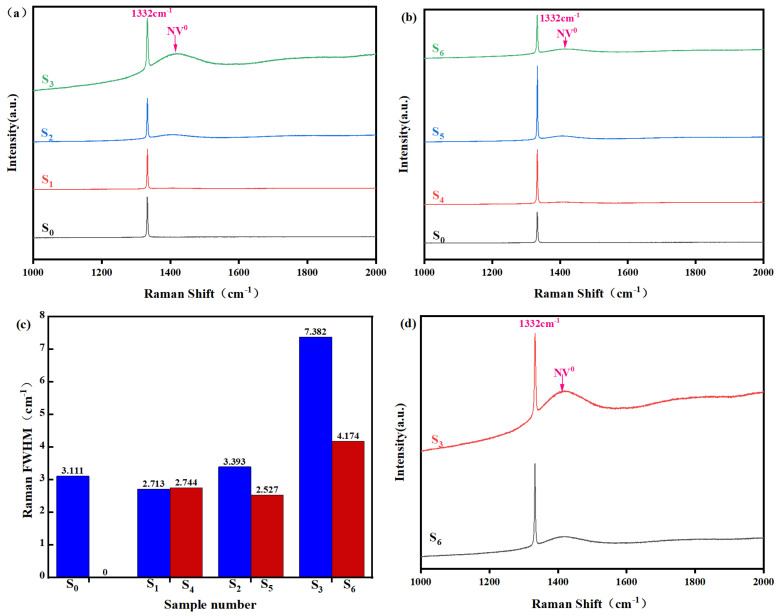
Raman spectra of diamond films with different nitrogen doping concentrations, (**a**) no carbon dioxide introduced and (**b**) adding carbon dioxide and their (**c**) their Raman FWHM. (**d**) Comparison of Raman spectra with high-concentration nitrogen with or without added Carbon dioxide.

As shown in Figure 2a, the Raman spectra of samples prepared under different parameters all exhibit a strong characteristic peak at 1332 cm^−1^. The absence of detectable D or G bands around 1350 cm^−1^ and 1580 cm^−1^ [29], which are indicative of amorphous carbon or graphitic impurities, confirms the high phase purity of the diamond. Furthermore, with the introduction of nitrogen, the spectra show no significant impurity peaks other than the emerging feature at 1425 cm^−1^, attributed to the NV^0^ center [15]. This further attests to the high purity and good crystallinity of the synthesized product. Close examination of Figure 2a reveals that the NV^0^ peak is not distinctly discernible at low nitrogen concentrations. However, its intensity becomes progressively more pronounced with increasing nitrogen flow. This trend is directly linked to the formation mechanism of nitrogen-vacancy (NV) centers: the incorporation of nitrogen leads to the formation of substitutional nitrogen atoms, which can subsequently bind with adjacent lattice vacancies to create NV centers. A higher nitrogen concentration in the process gas thereby increases the population of these NV centers, resulting in the enhanced visibility of their characteristic Raman signature [26]. The influence of CO_2_ introduction is revealed in the Raman spectra of Figure 2b, where a marked suppression of the NV^0^ peak at 1425 cm^−1^ is observed across all nitrogen levels. This is explained by the etching action of OH radicals, produced from CO_2_, on the nitrogen-vacancy (NV) centers. Figure 2d highlights that this etching effect is strongest in films grown with high nitrogen influx. Meanwhile, the crystalline quality, as probed by the FWHM of the diamond Raman peak (Figure 2c), shows a non-monotonic dependence on nitrogen concentration. The fitted FWHM values of 3.111, 2.713, 2.744, 3.393, 2.527, 7.382, and 4.174 first decline and then rise. This progression suggests that low-level nitrogen doping enhances crystallinity, whereas further increasing the nitrogen concentration ultimately compromises the crystalline perfection of the diamond film. Furthermore, at nitrogen concentrations above 24.93 ppm, the fitted full width at half maximum (FWHM) values for samples grown with CO_2_ introduction are consistently lower than those without CO_2_. This result indicates that the etching effect significantly reduces crystal defects, which are primarily caused by lattice expansion induced by nitrogen incorporation. The improvement in both bulk crystal and surface quality is particularly evident at high nitrogen concentrations. 

The residual stress in the diamond film can be determined based on the peak shift in the Raman spectrum, specifically by measuring the difference between the observed diamond peak position and the standard stress-free value of 1332 cm^−1^ (2) [30]

σ = −1.08 cm^−1^/Gpa (ν_1_ − ν_0_) (2)

where −1.08 cm^−1^/GPa is the Raman stress factor for diamond, ν_1_ denotes the measured Raman shift under stress, and ν_0_ = 1332 cm^−1^ represents the Raman shift of stress-free diamond.

Table 3 lists the obtained Raman peak positions and the corresponding residual stresses for the synthesized samples. For the characteristic diamond peak at 1332 cm^−1^, samples S_0_ to S_3_ all exhibit a positive shift from the standard position. This positive shift indicates the presence of compressive stress within the diamond lattice [31]. The residual stresses calculated accordingly are 0.69 GPa, 1.57 GPa, 1.27 GPa, and 1.35 GPa, respectively. It is evident that the residual stress in nitrogen-doped diamond crystals (S_1_–S_3_) is significantly higher than that in the undoped sample (S_0_). When a trace amount of Carbon dioxide was introduced during film growth, the corresponding doped diamond crystals (S_4_–S_6_) exhibited residual stresses of 1.35 GPa, 1.31 GPa, and 0.98 GPa, respectively, all of which demonstrate a certain degree of reduction compared to their Carbon dioxide-free counterparts with similar doping. The residual stress primarily originates from two sources: first, thermal stress resulting from the difference in the coefficient of thermal expansion (CTE) between the substrate and the diamond film; and second, intrinsic stress induced by the lattice mismatch between the dopant atoms and carbon atoms [32]. Nitrogen doping introduces substitutional nitrogen atoms that form C–N bonds during growth. Since the C–N bond differs in length from the original C–C bond, this substitution induces local lattice expansion, consequently leading to an increase in intrinsic stress and localized lattice distortion [33]. The observed stress reduction upon the introduction of trace Carbon dioxide is attributed to the etching effect of OH radicals on the crystal surface. This etching process helps to partially relieve lattice strain and suppress excessive lattice expansion, a mechanism consistent with the Raman FWHM results presented in Figure 2c. However, the residual stress in Carbon dioxide-co-doped samples remains higher than that in the undoped sample, indicating that the lattice expansion effect induced by nitrogen doping still dominates the overall stress state.

**Figure 3:**
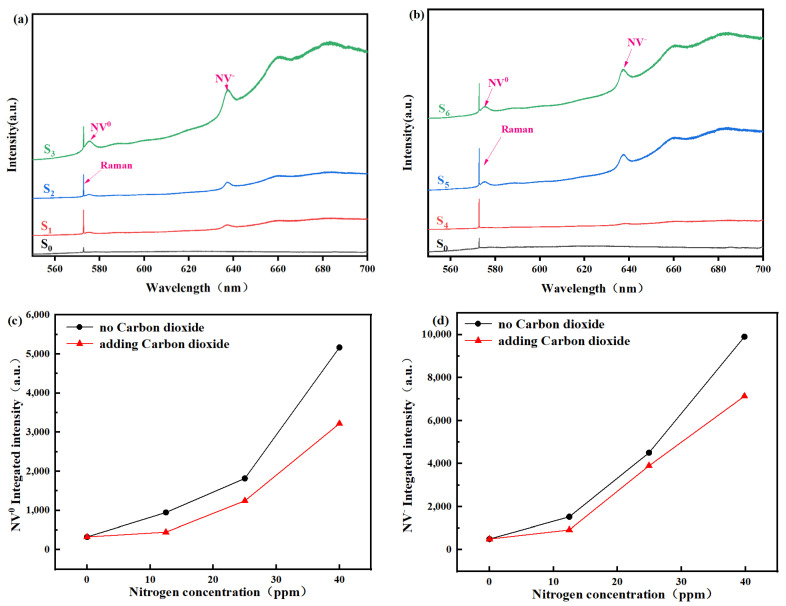
Photoluminescence spectra of diamond films with different nitrogen doping concentrations, (**a**) no carbon dioxide and (**b**) adding carbon dioxide, and the peak area of (**c**) NV^0^ and (**d**) NV^−.^

Figure 3a presents the photoluminescence (PL) spectra of diamond films prepared with varying nitrogen concentrations. A sharp characteristic diamond Raman peak is evident at 572 nm in the PL spectra of all films, regardless of the nitrogen concentration [15]. When the nitrogen concentration was 0 ppm, sample S0 exhibited the flattest PL baseline, with no detectable emission peaks corresponding to the NV^0^ center at 575 nm or the NV^−^ center at 637 nm. In contrast, as the nitrogen concentration increased to 12.48 ppm, a significant enhancement in the intensity of the characteristic diamond peak was observed, indicating improved film crystallinity. A concomitant rise in the spectral baseline was also noted, suggesting a slight increase in film impurities [33]. The luminescence peak for the NV^0^ center at 575 nm remains faint, whereas the NV^−^ peak at 637 nm has intensified markedly. When the nitrogen concentration reaches 24.93 ppm, the spectral baseline exhibits a slight increase. Concurrently, the relative intensity of the characteristic diamond Raman peak shows a minor enhancement, accompanied by a notable strengthening of the NV-related luminescence peaks. As the nitrogen concentration is further elevated to 39.84 ppm, the relative intensity of the diamond Raman peak experiences a slight decrease. Meanwhile, both the spectral baseline and the intensity of NV-related luminescence peaks surge significantly, demonstrating a substantial increase in nitrogen-related defects within the film. Furthermore, the integrated area of the respective luminescence peaks in the PL spectra is directly proportional to the concentration of NV^0^ and NV^−^ defects in the diamond film [33]. Figure 3b displays the photoluminescence (PL) spectra of diamond films grown with CO_2_ addition under varying nitrogen concentrations. The diamond Raman peak at 572 nm exhibits a similar trend of initial increase followed by a decrease, as observed in films without CO_2_. Similarly, the intensities of the NV^0^ peak at 575 nm and the NV^−^ peak at 637 nm increase with higher nitrogen concentrations. However, the rate of this increase is markedly lower compared to the samples without CO_2_, and this suppressive effect is more pronounced at higher nitrogen levels. These results demonstrate that the etching action by OH radicals, introduced via CO_2_, effectively reduces the incorporation of nitrogen impurities in the films, which is consistent with the Raman spectroscopy data in Figure 2. Furthermore, the integrated areas of the NV^0^ (575 nm) and NV^−^ (637 nm) luminescence peaks serve as indicators for the respective concentrations of these nitrogen-vacancy defects in the diamond film [34]. As demonstrated in Figure 3c,d, the concentration of nitrogen-vacancy (NV) defects exhibits a positive correlation with increasing nitrogen concentration. Furthermore, under conditions without CO_2_ introduction, the integrated areas of the luminescence peaks for both NV^−^ and NV^0^ centers in the prepared diamond films are consistently larger than the corresponding values from samples grown with CO_2_. 

**Figure 4:**
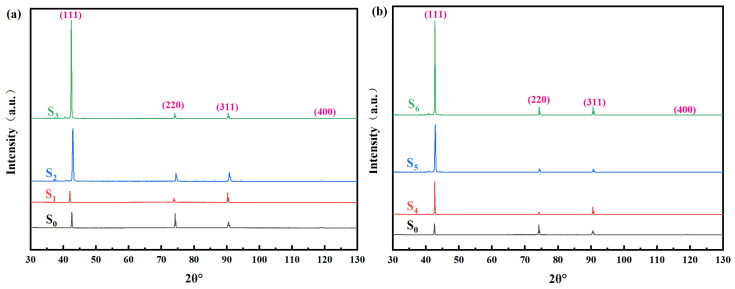
X-ray diffraction patterns (XRD) of diamond films with different nitrogen doping concentrations (**a**) with trace carbon dioxide introduced and (**b**) without carbon dioxide introduced, (**a**) with trace carbon dioxide introduced and (**b**) without carbon dioxide introduced.

Figure 4a,b presents the X-ray diffraction (XRD) patterns of diamond growth surfaces under different processing conditions, along with the relative intensities and corresponding variations in diffraction peaks from various crystal planes. The results show that the primary diffraction peaks of the as-grown diamond films within the 2θ range are located at 43.9°, 75.3°, 91.5°, and 119.7°, corresponding to the four main crystal planes: <111>, <220>, <311>, and <400>, respectively. It can be clearly observed that no distinct <400> diffraction peak is present in any of the samples. For the undoped sample S0 prepared at approximately 850 °C, the crystal orientation consists mainly of <111> and <220>, accompanied by a minor <311> orientation, while the overall crystal morphology appears relatively disordered. This indicates that the process parameters (temperature and nitrogen concentration) may be unfavorable for the nucleation and growth of the <100> crystal plane in diamond. Furthermore, with increasing MN flow rate (i.e., nitrogen concentration), the intensities of the <220> and <311> diffraction peaks decrease significantly, whereas the <111> diffraction peak intensity shows a marked enhancement. This suggests that at a growth temperature around 850 °C, the introduction of trace nitrogen can suppress the growth of other crystal planes, thereby promoting the preferential growth of the <111> orientation. In addition, as shown in Figure 4b, after the introduction of carbon dioxide, the reduction in the intensities of the <220> and <311> diffraction peaks becomes more pronounced. Moreover, under the same nitrogen concentration, the peak intensities of the films with CO_2_ addition are consistently lower than those without.

### 3.2. Microstructural Analysis

**Figure 5:**
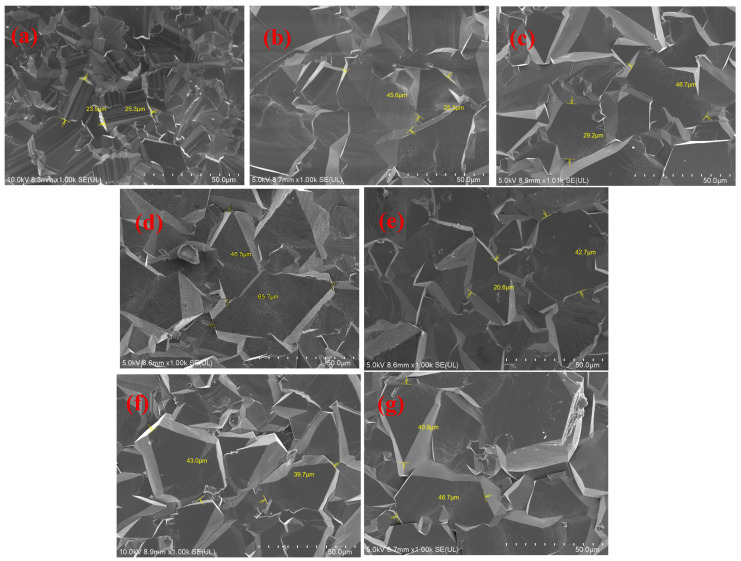
SEM images of the growth surfaces of samples (**a**) S_0_, (**b**) S_1_, (**c**) S_2_, (**d**) S_3_, (**e**) S_4_, (**f**) S_5_ and (**g**) S_6_ under different nitrogen concentrations.

The crystal structure of polycrystalline diamond films plays a decisive role in determining their thermal conductivity, mechanical strength, surface roughness, and optical properties. In such polycrystalline systems, the transport behavior of both photons and phonons is strongly influenced by microstructural features within the material, such as pores, secondary phase inclusions, impurity elements, and the presence of grain boundaries. To investigate the influence of different process parameters on the microstructure of free-standing diamond films, this section examines the surface morphology of nitrogen-doped diamond films before and after the addition of trace amounts of carbon dioxide. The lattice structure of the as-deposited diamond films was further studied using high-resolution electron microscopy. Figure 5 shows scanning electron microscopy (SEM) images of the growth surfaces of unpolished samples S0 to S6. As seen in Figure 5a, in the absence of nitrogen, the grains on the growth surface of S0 are composed of dense and uniform pyramid-like and prismatic particles with sizes ranging approximately from 23.5 μm to 25.3 μm. From Figure 5b–d, it can be observed that at a growth temperature of approximately 850 °C, with increasing nitrogen flow rate, the grains on the growth surfaces of S_1_–S_3_ become significantly larger compared to the undoped sample. Most of the exposed grain facets are pyramid-like particles with a predominant (111) orientation. This transition toward larger grain size can be attributed primarily to the step-bunching phenomenon induced by accelerated growth rates due to the introduction of nitrogen [35]. Meanwhile, as the nitrogen concentration increased progressively from 12.48 ppm to 39.84 ppm, the grain size of the diamond particles also exhibited a continuous enlargement, growing from approximately 23.5–45.6 μm for sample S_1_ to about 46.5–65.7 μm for S_3_. However, as shown in Figure 5d, excessively large grains can lead to an insufficiently dense microstructure, resulting in the formation of residual micropores or intergranular pores along the grain boundaries. According to reports [36], for polycrystalline optical ceramics, such microscopic or intergranular pores can lead to degradation of optical performance. Furthermore, as shown in Figure 5e,f, the grain sizes of diamond particles on the growth surfaces of S4–S6 are 20.6–42.7 μm, 39.7–43.0 μm, and 40.9–46.7 μm, respectively. Compared to samples S1–S3 at the same nitrogen concentrations, these values indicate a certain degree of reduction in both grain size and intergranular porosity. This suggests that the etching effect induced by the introduction of carbon dioxide effectively suppresses the degradation of film quality caused by excessive nitrogen doping, which is consistent with the decrease in Raman FWHM observed in Figure 2.

### 3.3. Thermodynamic Properties of Diamond Materials

Assuming transient heat transfer within the sample and neglecting heat loss during testing, a laser pulse was emitted to irradiate the bottom surface of the substrate. The subsequent absorption of heat led to a temperature increase in the sample, and the corresponding temperature rise on the top surface was measured. The thermal diffusivity of samples S_0_–S_6_, as listed in Table 4, was determined based on the time required for the temperature rise to reach half of its maximum value. The thermal conductivity of diamond was calculated using the formula [37,38]:

K = α × ρ × Cp(3)

In this formula, α represents the thermal diffusivity under different growth conditions, while ρ and Cp denote the density of diamond (3.52 g·cm^−3^) and the specific heat capacity (0.519 J·g^−1^·K^−1^).

**Figure 6:**
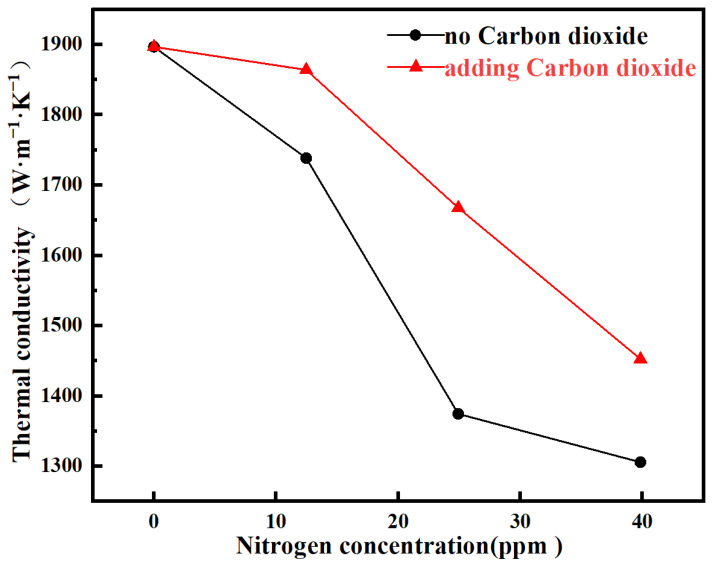
Thermal conductivity of diamond films under different nitrogen gas flow rates before and after adding oxygen.

Figure 6 shows the thermal conductivity of samples prepared under different processes as a function of nitrogen concentration. It can be clearly observed that the thermal conductivity of the films decreases with increasing nitrogen concentration. This phenomenon can be attributed to the following mechanism: The high thermal conductivity of diamond primarily originates from the efficient propagation of lattice vibrations (i.e., phonons). Any defects that disrupt the perfection of the crystal lattice can scatter phonons, thereby reducing thermal conductivity. The introduction of nitrogen exacerbates phonon scattering mainly through the formation of complex defects and the induction of internal stress. Nitrogen atoms in diamond tend to combine with other defects, such as vacancies, forming more complex defect centers (e.g., nitrogen-vacancy color centers). These defects simultaneously introduce internal stress into the lattice, further disturbing its periodicity and intensifying phonon scattering [39]. We aim to elucidate the synergistic effect introduced by trace CO_2_ incorporation under fixed nitrogen doping levels. The specific data analysis is as follows: compared to the nitrogen-only doped sample S1, the thermal conductivity of the co-doped sample S_4_ increased by 125.47 W·m^−1^·K^−1^; compared to sample S_2_, sample S_5_ increased by 293.17 W·m^−1^·K^−1^; and compared to sample S_3_, sample S_6_ increased by 146.79 W·m^−1^·K^−1^. These results indicate that the introduction of trace CO_2_ co-doping on top of nitrogen doping can significantly enhance the thermal conductivity of the films, with an improvement ranging approximately between 125 and 300 W·m^−1^·K^−1^. Particularly under low nitrogen concentration (12.48 ppm), the degradation in thermal conductivity induced by nitrogen doping was almost completely suppressed. This phenomenon can be attributed to the fact that introduced CO_2_ effectively etched the film during growth, thereby suppressing the formation of complex defect centers such as NV centers caused by nitrogen doping. The elimination of such defects mitigates excessive lattice expansion and associated internal stress, which is consistent with the observations from Raman and photoluminescence spectroscopy discussed previously.

## 4. Conclusions

1. Advantage of introducing trace amounts of carbon dioxide: This study successfully demonstrates that the co-doping strategy utilizing N_2_ and CO_2_ during MPCVD growth is a highly effective method for fabricating high-quality, optical-grade polycrystalline diamond films. This approach ingeniously leverages the dual functions of N_2_ in promoting growth rate and CO_2_ in purifying the crystal lattice.
2. Performance Optimization: Employing this method enabled the attainment of diamond films featuring high thermal conductivity (1863.94 W·m^−1^·K^−1^) and a highly <111>-textured morphology, while maintaining a high growth rate (~3–4 µm/h) and effectively mitigating defects introduced by nitrogen impurities.
3. Defect Control Mechanism: The introduction of CO_2_, through its etching action, significantly reduces the concentration of complex defects such as NV colour centres and alleviates lattice strain. This mechanism is identified as the fundamental reason for the observed enhancements in both thermal conductivity and optical properties.
4. Application Prospect: This work provides an effective solution to the long-standing challenge of reconciling crystal quality with performance in nitrogen-doped diamond. The fabricated ultra-low nitrogen-doped diamond films exhibit broad application prospects in fields such as high-efficiency thermal management.

## Figures and Tables

**Table 1:** Experiment parameters of deposition processes.

Samples	H_2_/sccm	CH_4_/sccm	MN/sccm	CO_2_/sccm	Power/kw	Pressure/Torr	Temperature/°C
S_0_	400	12	0	0	3.8	100	850 ± 10
S_1_	400		0.5	0			
S_2_	400		1	0			
S_3_	400		1.6	0			
S_4_	400		0.5	1			
S_5_	400		1	1			
S_6_	400		1.6	1			

MN:H_2_-N_2_ mixture gas with a nitrogen content of 1%.

**Table 2:** Characteristics parameters of thickness and growth rates of the diamond samples.

Schemes	Thickness/μm	Growth Rate μm/h
S_0_	45	1.125
S_1_	84	2.100
S_2_	111	2.775
S_3_	139	3.475
S_4_	95	2.375
S_5_	125	3.125
S6	150	3.750

**Table 3:** Raman shifts and residual stress in the diamond samples.

Samples	Raman Shift/cm^−1^	Residual Stress/GPa
S_0_	1332.64	−0.69
S_1_	1333.45	−1.57
S_2_	1333.18	−1.27
S_3_	1333.25	−1.35
S_4_	1333.21	−1.31
S_5_	1333.08	−1.17
S_6_	1332.91	−0.98

**Table 4:** Thermal conductivity of diamond samples.

Samples	Thermal Diffusivity/mm^2^·S	Thermal Conductivity/W·m^−1^·K^−1^
S_0_	1038.34	1896.93
S_1_	951.61	1738.47
S_2_	752.39	1374.52
S_3_	714.79	1305.84
S_4_	1020.29	1863.94
S_5_	912.86	1667.69
S_6_	795.14	1452.63

## Data Availability

The original contributions presented in this study are included in the article/Supplementary Materials. Further inquiries can be directed to the corresponding authors.

## Supplementary Materials

The following supporting information can be downloaded at: https://www.mdpi.com/article/10.3390/ma19010183/s1.
