# Supplementary figures and images for: Enhancing the Quality of Diamond Film Growth Through the Synergistic Addition of Nitrogen and Carbon Dioxide
Source: Materials (Basel). 2026 Jan 4;19(1):183. doi: 10.3390/ma19010183 (PMC12787062; doi:10.3390/ma19010183)

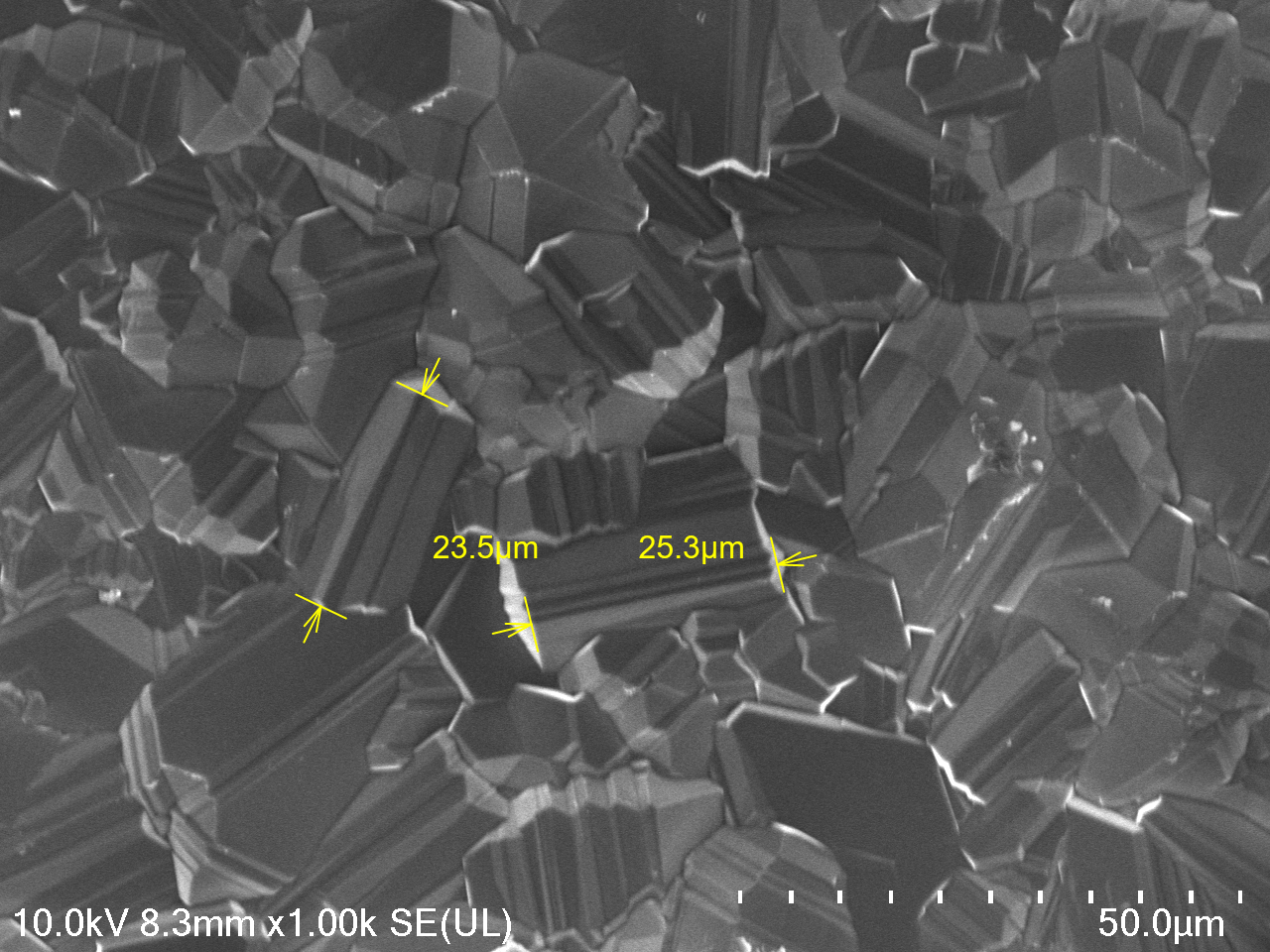

Supplement: Supplementary file 1 [file materials-19-00183-s001.zip › SEM/Different nitrogen flow rates/0N2_0093.tif]

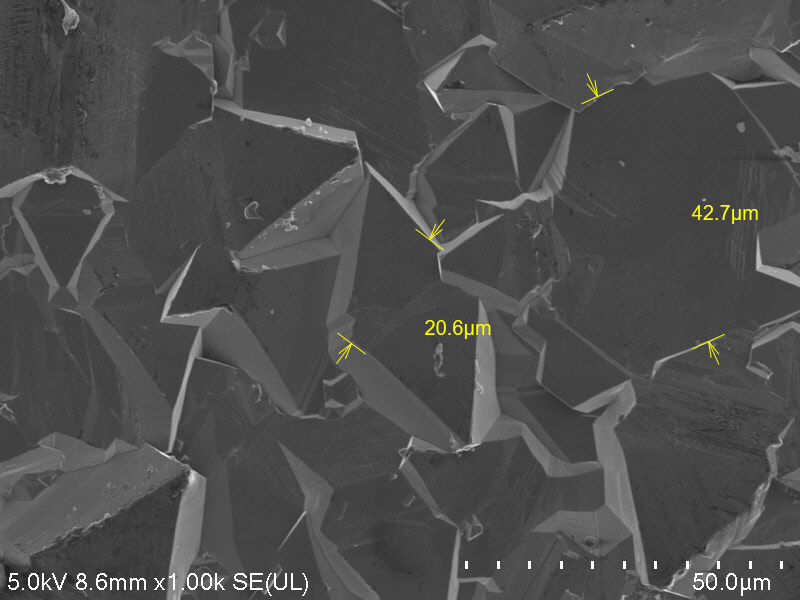

Supplement: Supplementary file 1 [file materials-19-00183-s001.zip › SEM/Different nitrogen flow rates/12.5PPM+1SCCCMO2_0029.jpg]

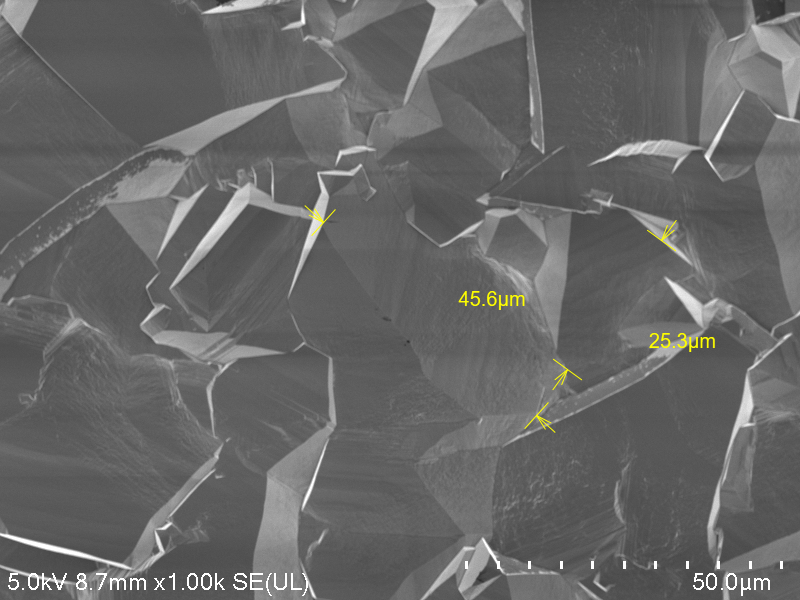

Supplement: Supplementary file 1 [file materials-19-00183-s001.zip › SEM/Different nitrogen flow rates/12.5PPM_0073.tif]

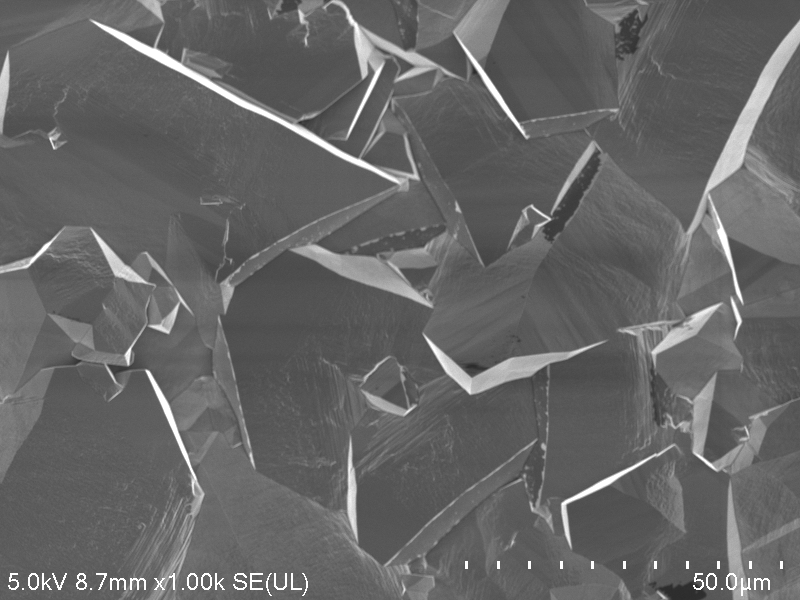

Supplement: Supplementary file 1 [file materials-19-00183-s001.zip › SEM/Different nitrogen flow rates/12.5PPM_0074.tif]

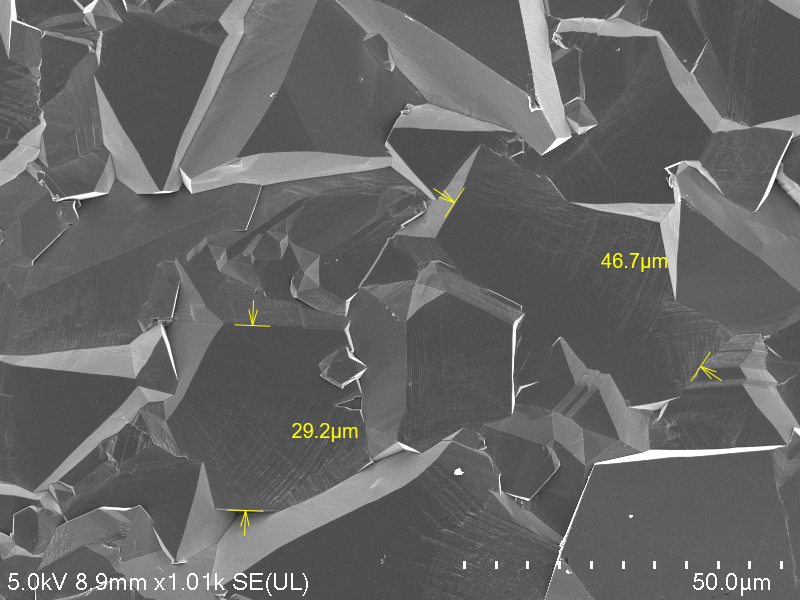

Supplement: Supplementary file 1 [file materials-19-00183-s001.zip › SEM/Different nitrogen flow rates/25ppm_0018.tif]

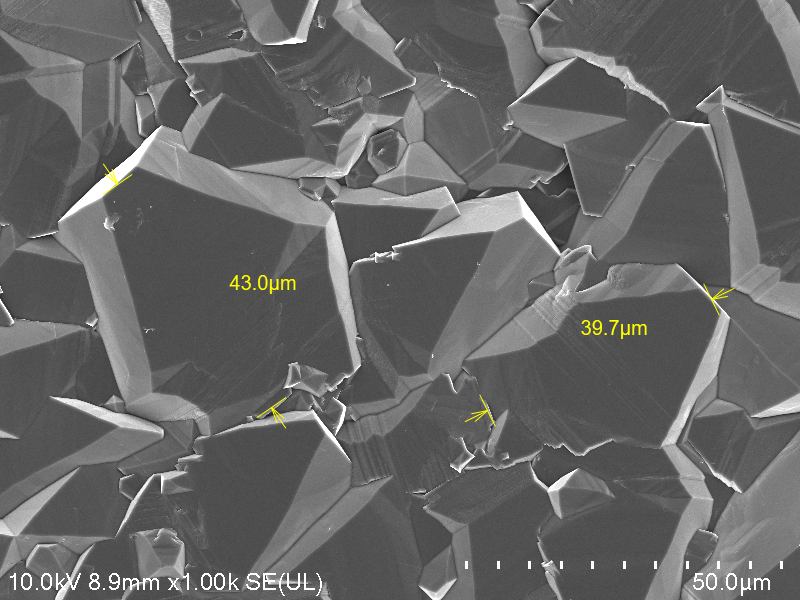

Supplement: Supplementary file 1 [file materials-19-00183-s001.zip › SEM/Different nitrogen flow rates/25PPMN2+1SCCMO2_0019.tif]

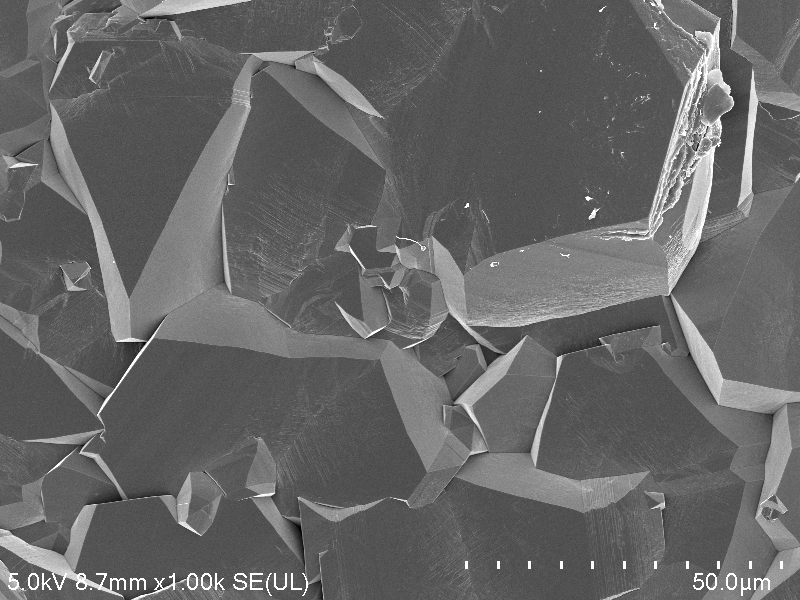

Supplement: Supplementary file 1 [file materials-19-00183-s001.zip › SEM/Different nitrogen flow rates/40PPM N21SCCMO2_0097-.tif]

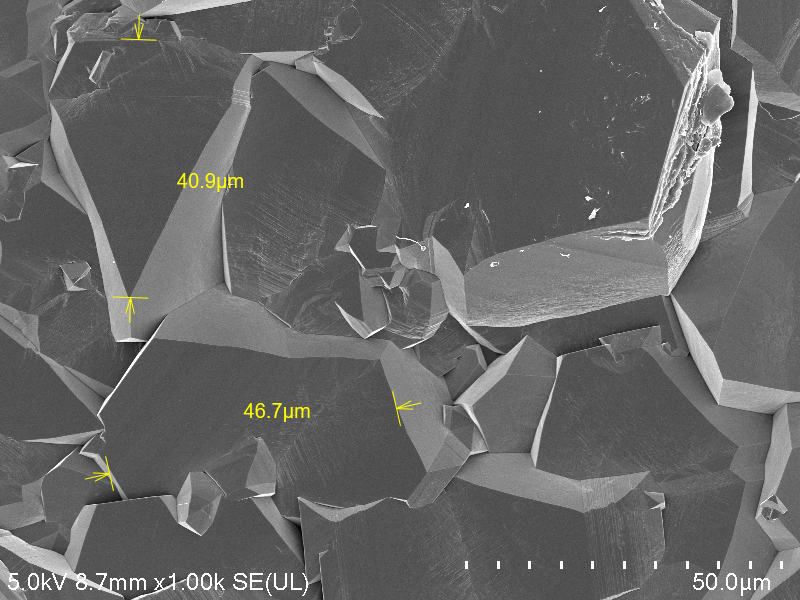

Supplement: Supplementary file 1 [file materials-19-00183-s001.zip › SEM/Different nitrogen flow rates/40PPM N21SCCMO2_0097.tif]

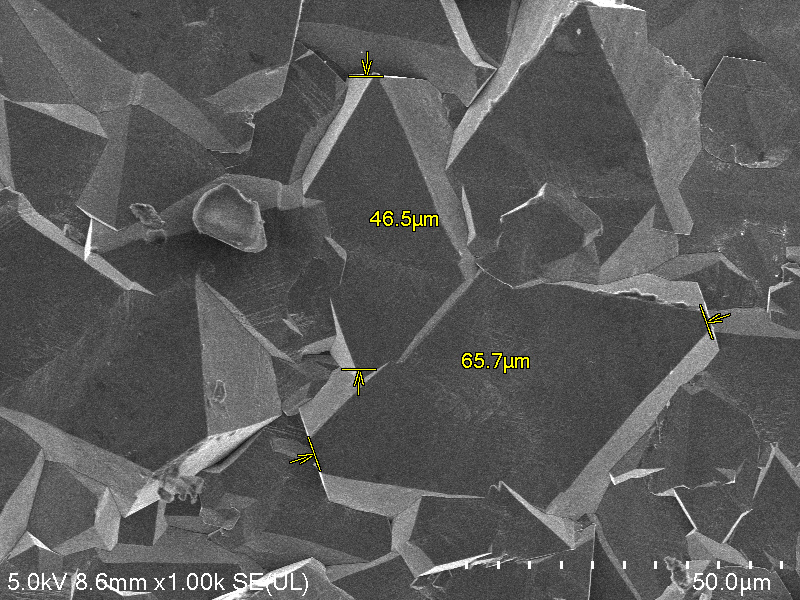

Supplement: Supplementary file 1 [file materials-19-00183-s001.zip › SEM/Different nitrogen flow rates/40ppmN2_0012.jpg]
